# Clustered embedding using deep learning to analyze urban mobility based on complex transportation data

**DOI:** 10.1371/journal.pone.0249318

**Published:** 2021-04-20

**Authors:** Sung-Bae Cho, Jin-Young Kim

**Affiliations:** 1 Graduate School of Artificial Intelligence, Yonsei University, Seoul, South Korea; 2 Department of Computer Science, Yonsei University, Seoul, South Korea; Shenzhen University, CHINA

## Abstract

Urban mobility is a vital aspect of any city and often influences its physical shape as well as its level of economic and social development. A thorough analysis of mobility patterns in urban areas can provide various benefits, such as the prediction of traffic flow and public transportation usage. In particular, based on its exceptional ability to extract patterns from complex large-scale data, embedding based on deep learning is a promising method for analyzing the mobility patterns of urban residents. However, as urban mobility becomes increasingly complex, it becomes difficult to embed patterns into a single vector because of its limited capacity. In this paper, we propose a novel method for analyzing urban mobility based on deep learning. The proposed method involves clustering mobility patterns and embedding them to capture their implicit meaning. Clustering groups mobility patterns based on their spatiotemporal characteristics, and embedding provides meaningful information regarding both individual residents (i.e., personalized mobility) and all residents as a whole, enabling a more effective analysis of mobility patterns. Experiments were performed to predict the successive points of interest (POIs) based on transportation data collected from 1.5 million citizens in a large metropolitan city; the results demonstrate that the proposed method achieves top-1, 3, and 5 accuracies of 73.64%, 88.65%, and 91.54%, respectively, which are much higher than those of the conventional method (59.48%, 75.85%, and 80.1%, respectively). We also demonstrate that the proposed method facilitates the analysis of urban mobility through arithmetic operations between POI vectors.

## 1. Introduction

Based on the rapid growth of the Internet of Things technologies, including 5G, global positioning system (GPS) and smart cards, massive numbers of trajectories are being generated continuously by various sources [1]. In particular, in urban areas, mobility is relatively complex based on the scale of cities. In addition to the popularity of ubiquitous sensing and intelligent transportation systems, unprecedented mobility data have been gathered by exploiting a variety of mobile devices, such as smartphones and on-board GPSs, as well as automatic fare collection devices that are widely deployed in urban transit systems such as subways, buses, and taxies [2]. Mobility patterns can be defined as the combination of many elements and their interactions [3]. It is complicated to identify governing factors that encompass all types of mobility patterns; therefore, various simulations using agent-based models have been performed [4].

Based on this background, research on large-scale and reliable mobility pattern analysis has become a hot topic because urban mobility plays a crucial role in the growth, employment, and sustainable development of a city [5]. Emerging big data and related research can effectively augment data availability and enrich the utility of data, meaning that various services can be facilitated by predicting destinations based on analyzed mobility patterns [5, 6]. For example, by analyzing and predicting mobility patterns, useful information such as personalized services and future traffic flow predictions can be provided.

Based on multilevel and multi-source big geospatial data, significant research efforts have been devoted to approximating spatiotemporal urban mobility patterns using GPS data [7], smart card records [8], mobile positioning data [9], and other data. Additionally, several studies have focused on characterizing urban/human mobility patterns and have attempted to derive universal laws [10–12]. Previous works have identified and leveraged the strong associations between urban mobility and additional information such as land use [13], spatial structure [14], building environments [15], and personal information [16].

Recently, a method for embedding large amounts of sequence information was developed to enhance the analysis of the movement patterns of urban residents [16–26]. Regarding methods for analyzing mobility patterns, matrix factorization [17, 18], deep learning models based on recurrent neural networks (RNNs) [19–22], and RNNs with preference information [16, 23–26] are widely used. In previous works, because individual mobility patterns have been embedded in single vectors, it has been difficult to capture all of the information related to increasingly complex mobility patterns. In particular, if all the residents shared only one embedding vector, the majority movement patterns from a location “A” to a particular destination “B” could be embedded, and those from a location “A” to other destinations would be ignored.

This issue can be resolved naively by calculating embedding vectors for every resident (or data), but this leads to major issues in terms of tremendous numbers of embedding models and small amounts of relevant data per individual. In this paper, we propose a mobility pattern analysis method consisting of clustering similar mobility patterns and embedding the resulting clusters. Because the perception of a location may be different for different residents (for example, a shopping mall may be a leisure place for customers, but a workplace for employees), our clustering method defines mobility patterns by considering spatiotemporal characteristics. Additionally, our embedding method obtains mobility patterns not only for each resident (i.e., personalized mobility), but also for all residents as a whole to analyze mobility patterns more effectively.

We collected real-world data to verify the proposed method. Approximately 100 million large-scale transportation data were collected in Seoul, South Korea over six months. These mobility data were collected from smart cards for subways and buses, and consist of log records such as time, user IDs, and station IDs from when the smart cards were used. There are approximately 1.5 million users and 16,000 points of interest (POIs). Additional details regarding the collected data are discussed in Section 2. The main contribution of this paper is summarized as follows:

- We propose a novel method to embed and analyze the urban mobility patterns with large number of movement data in metropolitan cities.
- This addresses the problem of sharing the POI embedding vector with all residents, resulting in ignoring the minor patterns.
- The personalized embedding method with clustering technique can cope with the large amount of the embedding vectors.
- Experiments with 1.5 million citizens data are conducted to verify the proposed method.

The remainder of this paper is organized as follows. In Section 2, we discuss the details of the collected real-world mobility data. The proposed method for mobility analysis is detailed in Section 3 and its performance is verified in Section 4. Section 5 presents relevant works on mobility pattern analysis for comparison. Finally, we summarize our conclusions and discuss future work in Section 6.

## 2. Complex mobility in urban areas

**Fig 1:**
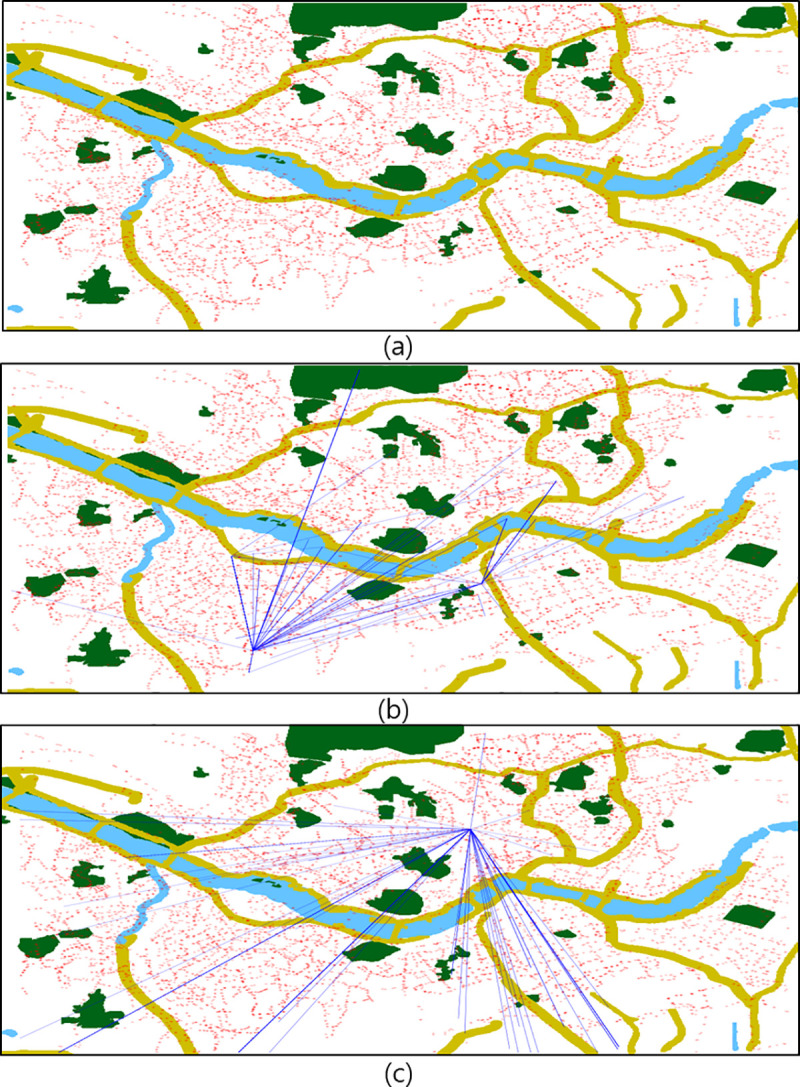
Maps representing the collected mobility data: (a) map with stations, (b) map with mobility data from one user, and (c) map with the mobility data from one station.

The mobility data from Seoul were collected from transportation cards called smart cards from January 2018 to June 2018. These cards are similar to Oyster cards in the United Kingdom, Metro cards in New York, PASMO cards in Tokyo, and Opal cards in Sydney. Approximately 100 million movement sequence data were collected from approximately 1.5 million residents. The attributes of the collected data are listed in Table 1. Fig 1 illustrates the complexity of the data, where part (a) presents a map with stations indicated by red dots, respectively. Parts (b) and (c) present the mobility data from one user and the mobility data from one station, respectively.

**Table 1:** Summary of the attributes of the collected data.

Name	Description	Name	Description
TRCR_NO	Index of the transportation card	BASE_AMT	Basic rate
TRNS_TRD_TRNC_ID	Index of the traffic transaction	ADD_AMT	Surcharge
BASE_DT	First boarding date	FIRST_ON_DTM	First boarding time
CLOS_DT	Last boarding date	LAST_OFF_DTM	Last drop off time
WEEK_DAY	Day of the week	FIRST_STN_ID	First station ID
STN_CNT	Number of stations	LAST_STN_ID	Last station ID
TOT_AMT	Total fare	TRD_SEQ_CD	Transaction sequence number
TOT_ELAPED_TM	Total elapsed time	SEX_CD	Sex information of user
TRAN_CNT	Number of transfers	BIRTH_YMD	Birth information of user

The total number of POIs that can be reached by users is 16,000 and the facility information for each POE is classified as “education,” “shopping,” “entertainment,” “public institution,” “medical care,” “meal,” or “other.” We excluded approximately 20 million based on missing entries for users who did not input their information. Despite this exclusion, the size of the dataset used in this study is very large compared to those used in previous works [17–19, 23, 27], as shown in Table 2.

**Table 2:** Comparison of datasets.

Dataset	# Users	# Movement seq.	# POIs	Period
NYC [19]	975	64,702	4,722	**10 months**
Yelp [27]	11,564	11,564	18,683	-
Forsquare1 [17]	3,571	744,055	28,754	4 months
Gowalla [18]	3,420	556,453	**33,578**	7 months
Forsquare2 [23]	2,321	194,108	5,596	4 months
Our work	**1,561,147**	**118,708,678**	16,080	6 months
Our work (Preprocessed)	**74,241**	**18,536,898**	16,080	6 months

**Fig 2:**
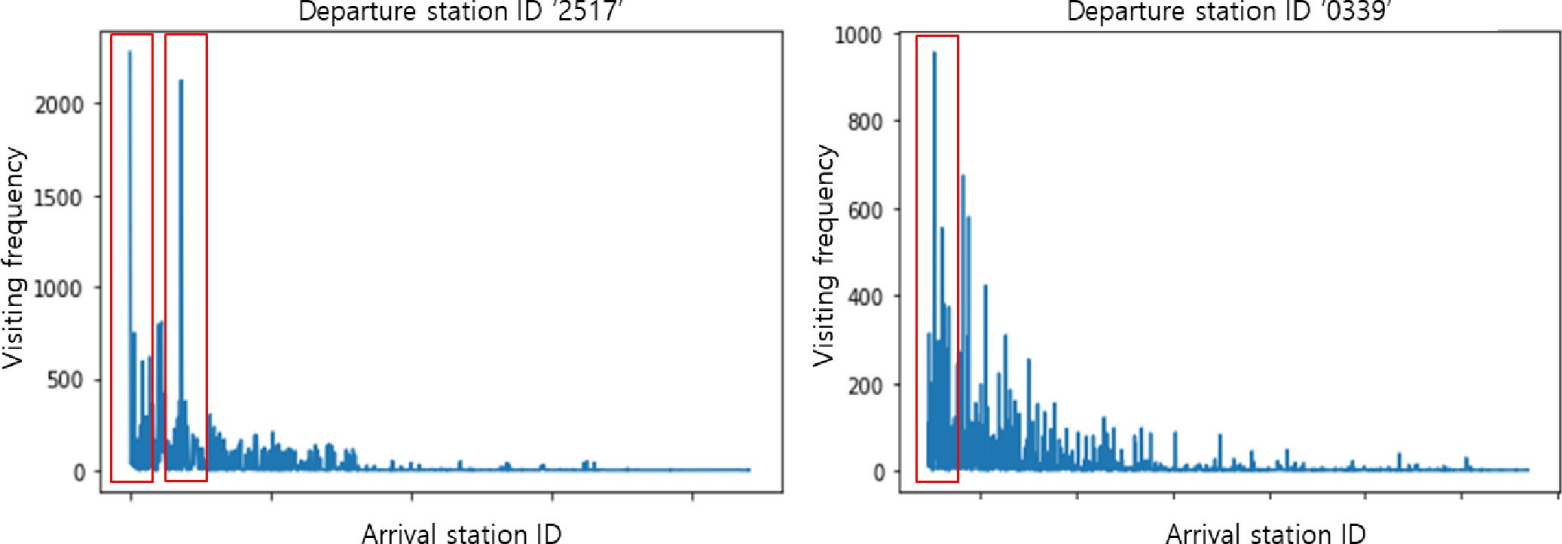
Numbers of data for stations that can be reached from departure stations (a) “2517” and (b) “0339”.

Fig 1 indicates that conventional embedding methods based on statistics do not work well because mobility with respect to stations and individuals is complex and variable. As shown in Fig 2, the departure station with an ID of “2517” has two arrival stations with high frequencies, resulting in the risk that other stations could be ignored when mobility patterns are analyzed.

## 3. Embedded mobility patterns

**Fig 3:**
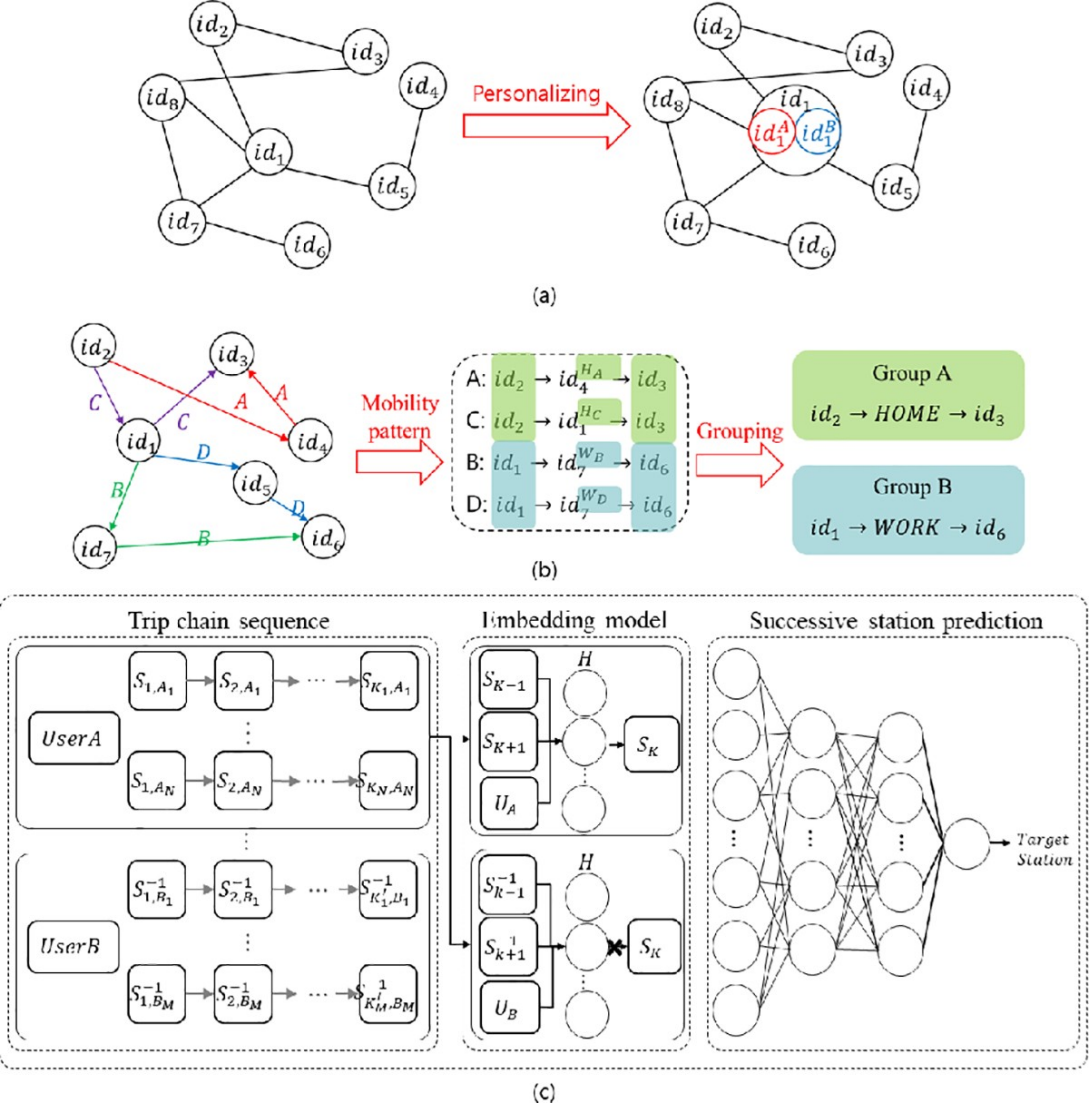
Overall process for (a) personalizing POIs, (b) clustering similar mobility patterns, and (c) embedding mobility vectors to predict successive POIs.

The overall architecture of the proposed method is illustrated in Fig 3. For personalized embedding, as shown in Fig 3(A), we generate a special sequence for each user by using the method described in Section 3.1. To cluster residents whose mobility patterns are similar, we define facility categories (e.g., “home”; *H*_*A*_, *H*_*C*_ and “workplace”; *W*_*B*_, *W*_*D*_) based on several rules and grouping residents based on their mobility patterns, as shown in Fig 3(B). This process is discussed in Section 3.2. Based on sequencing and clustering, we present a dual optimization process for embedding resident-aware mobility patterns in Section 3.3. Embedded vectors representing the characteristics of mobility pattern for a specific user (i.e., personalized embedding vectors) can be obtained from the proposed model, as shown in Fig 3(C). To verify the embedded mobility pattern vectors, we perform the prediction of successive POI IDs based on these vectors, as discussed in Section 3.4.

### 3.1 Personalized movement sequences

To train a model for embedding personalized mobility patterns, we generate a movement sequence before proceeding with the embedding process. The collected movement records can be chronologically ordered as $\left(id_{1}^{n_{1}},id_{2}^{n_{1}},\ldots ,id_{k_{n_{1}}}^{n_{1}}\right)$, where *id* is an index for station, *n*_*i*_ is an index of the transportation card regarded as resident *id* and $k_{n_{i}}$ is a length of the logged records for *n*_*i*_. Since the recorded movement has departure and destination points, *id*_2*j*−1_ and *id*_2*j*_ where $j=1,\ldots ,\lceil k_{n_{i}}/2\rceil$ are in “FIRST_STN_ID” and “LAST_STN_ID” shown in Table 1, respectively. Each resident can produce the movement sequences with difference length; active residents would have relatively long movement sequences while visiting many POIs, but a large amount of real data implies the movement patterns from most of the residents can be observed in a short period. Since we intend to embed and analyze the mobility patterns over a short period of time, we generate the sequences in every month. We generate a station ID that exists in one resident’s movement sequence, but does not appear in the sequences of other residents. This means that each POI is separated into several embedding vectors (as many as the number of residents who have visited the POI). Therefore, we can create perfectly personal mobility embedding vectors, the verification of which will be described in Section 4.

### 3.2 Clustering mobility patterns

**Fig 4:**
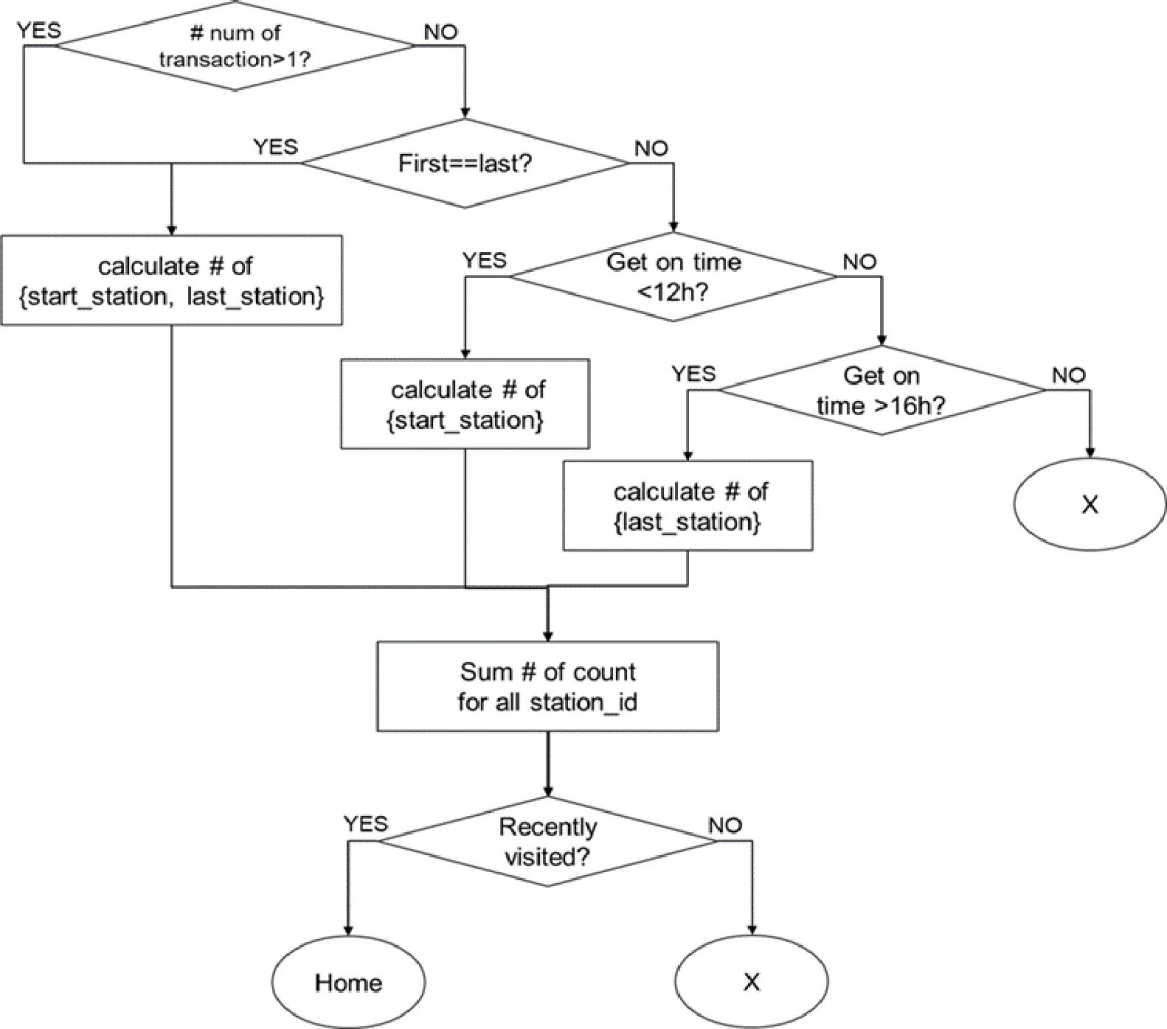
Rules for extracting the “home” class.

**Fig 5:**
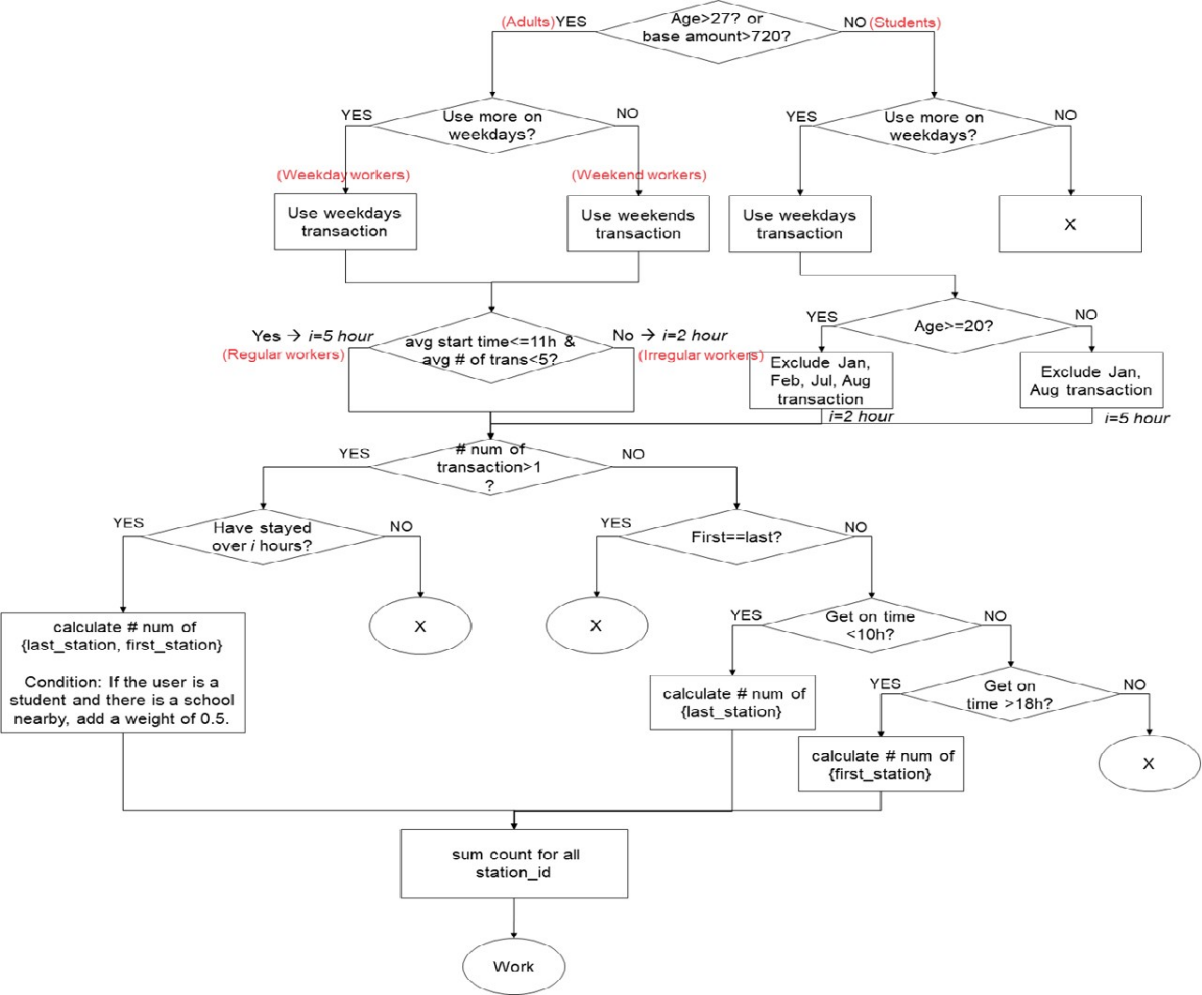
Rules for extracting the “workplace” class.

As mentioned previously, clustering can be useful for embedding complex patterns in a vector. To cluster residents with similar mobility patterns, movement patterns must be defined based on information regarding the POIs visited by residents. We categorize the characteristics of POIs into five classes of “home,” “workplace,” “third point,” “fourth point,” and “fifth point” based on previous statistical studies and mobility pattern analysis [28–33]. The rules for extracting the classes of “home” and “workplace” are presented in Figs 4 and 5, respectively. The classes of “third point,” “fourth point,” and “fifth point” are the most-visited places at which the user stayed for more than one hour and classified as “education,” “shopping,” “entertainment,” “public institution,” “medical care,” “meal,” and “other” according to the corresponding facility information.

Based on these results, we designed a method to cluster mobility patterns. First, the timeline is divided into four sections and the number of classes of departure POIs is checked. One continuous timeline would hinder from extracting appropriate patterns from unnecessarily large amount of information. We divide the time zone in which humans live in daily life into four sections according to Ma, et al. [29], which facilitates the extraction of routine features. The preliminary analysis of the collected data leads to the division of timeline as dawn (0~6 o’clock), morning (6~12 o’clock), afternoon (12~18 o’clock), and evening (18~24 o’clock), which have a ratio of 1.0: 1.13: 1.11: 1.03. We assign the most frequent POI class to the representative class in each time section. For example, if resident *A*’s representative class is home in the first section and workplace in the third section, with no information in the second and fourth sections. This resident’s cluster identification code is “home-other-workplace-other.” Algorithm 1 summarizes the process for generating a cluster ID.

**Algorithm 1.** Process for generating a cluster ID

**Input:** *POI*_*classes*_, *List*_*id*_

**Output:** *cluster*_*id*_

**for** *j* =*id*_1_,…,*id*_*N*_ **do**

    **for** *i* = *t*_1_,…,*t*_4_ **do**

        *C*_*p*_ = *Count*(*POI*_*classes*_, *history*(*j*,*i*))

      *cluster*_*id*_ = *concatenate*(*cluster*_*id*_,*B*(*C*_*p*_))

  **end for**

**end for**

**return *cluster***_***id***_

where *t*_*k*_ is the *k*^*th*^ time section, *history*(*j*,*i*) represents the function to get the mobility history for user with id *j* in time section *i*, *Count*(⋅,⋅) means the mapping from list of POI types and user *j*’s history to the list of frequency for each POI type, and *B*(⋅) is the operation for extracting the most frequent POI class.

### 3.3 Embedding mobility patterns via dual optimization

As mentioned previously, mobility pattern embedding with personalization is more efficient than other methods for analyzing mobility patterns. Each resident’s movement sequence is used to learn an embedding vector. Prior to the embedding process, we create a single basis vector representing the movement sequence, which is defined as a resident vector. If a resident has one or more corresponding movement data, then that resident’s movement sequence shares the same resident vector. During the training process for the proposed method, the current mobility pattern vector is learned similarly to the embedding vector of the previous mobility pattern, next POI, and resident vector, as indicated in Eq (1).

Let $S_{k,A_{j}}$ be the *k*^th^ place in the *j*^th^ movement sequence of resident *A*. Because we have a movement sequence for up to six months, the maximum value of *j* is six. Let *R*_*A*_ be the resident vector for resident *A* in the following equations.

$$L_{POI}=P(S_{k,A_{j}}|S_{k-1,A_{j}},S_{k+1,A_{j}},R_{A})=\exp\left(v(S_{k-1,A_{j}},S_{k+1,A_{j}},R_{A})\cdot (s_{k,A_{j}})\right)$$ (1)

$$v\left(S_{k-1,A_{j}},S_{k+1,A_{j}},R_{A}\right)=\frac{S_{k-1,A_{j}}+S_{k+1,A_{j}}+R_{A}}{3}$$ (2)

Our goal is to find an optimal vector for $S_{k,A_{j}}$ that maximizes the value of Eq (1). The mobility embedding vector is learned such that the probability of a target with a given mobility pattern vector sequence and resident vector will be maximized. Eqs (1) and (2) define how to calculate this probability. By adding resident information, we can learn a mobility embedding vector that is associated with the target resident. Eq (1) is also used for the resident vector *R*_*A*_ while the personalized mobility vector for resident *A* is being trained. We use an exponential function to quantify this vector for probabilistic modeling, as shown in Eq (1). As a result of this training process, mobility embedding vectors that are related to each other are optimized to have a high probability of appearing together (i.e., high cosine similarity).

To learn personalized mobility embedding and resident vectors effectively, we minimize Eq (3) while maximizing Eq (1). If the mobility embedding and resident vectors for a resident *B* are given, then the probability of *S*_*k*,*A*_ appearing with these vectors should be low. Therefore, the mobility embedding vectors for different residents are far from each other. Eq (4) defines the objective function for learning not only personalized embedding vectors, but also resident vectors. *X* is known information regarding the movement sequence data of other residents. When the numerator is maximized and the denominator is minimized in Eq (4), the probability of the desired target will be learned with the greatest efficiency.

$$L_{User}=\sum_{B\neq A}P(S_{k,A}|S_{k-1,B_{j}},S_{k+1,B_{j}},U_{B})$$ (3)

$$\max P\left(S_{k,A}|X\right)=\frac{\max\;L_{POI}}{\min\;L_{User}}$$ (4)

### 3.4 Analysis of mobility patterns

To verify the embedded mobility pattern vectors, we constructed a successive POI prediction model, the inputs of which are mobility pattern embedding vectors. This model is composed of a fully connected network layer (FCN), as shown in Eq (5), with the LeakyReLU activation function, as shown in Eq (6). The final layer is a softmax layer, as shown in Eq (7). For each group, we construct an FCN and set the size of the last layer equal to the number of candidate successive POIs. The proposed model learns to output confidence scores for candidates and selects the POI with the highest value.

$$x^{l+1}=f_{\alpha }^{l}(W^{l}x+b^{l}),$$ (5)

$$f_{\alpha }(x)=\left\{ \begin{array}{l} x\;if\;x\geq 0\\ \alpha x\;otherwise \end{array}\right.,$$ (6)

$$f(target_{i})=\frac{e^{target_{i}}}{\Sigma_{j}e^{target_{j}}},$$ (7)

where *x*^*l*^ is the output of the *l*^*th*^ layer; *x*^0^ is the input; *x*^*L*^ is the output; *L* is the depth of the FCN; *W*^*l*^ is the weight of the *l*^*th*^ layer; *b*^*l*^ is the bias of the *l*^*th*^ layer, and $f_{\alpha }^{l}$ is the activation function of the *l*^*th*^ layer, which is the LeakyReLU function for 0≤*i*<*L* and softmax function for *i* = *L*.

We use categorical cross entropy as a loss function, which is calculated using Eq (8), where *p*(*j*) is the true probability distribution and *q*(*j*) is the predicted probability distribution.

$$H(p,q)=-\sum_{j}p(j)log\;(q(j))$$ (8)

## 4. Experimental results and discussion

### 4.1 Experimental settings

To verify the proposed method, we used the collected dataset described in Section 2. Several experiments were conducted to evaluate the proposed method. Our experiments consisted of predicting the next POI ID using the proposed model and the validation of embedding vectors. We compared the prediction results for ten repeated experiments with different pairs of methods (embedding and clustering methods). We considered two clustering methods and three embedding methods. Random embedding, no personalized embedding, and random clustering were considered as baseline models. The top-k accuracy metric was used to evaluate all methods.

Our objective is to construct a personalized mobility embedding vector that can be verified by predicting successive POIs. To evaluate the performance of our model, we calculated the top-k accuracy values for *k* = 1,3, and 5. The mean reciprocal rank (MRR) was used to verify the personalized embedding vectors. MRR evaluates how close an output is to a target, ordered by the probability of correctness, as follows [34]:

$$MRR=\frac{1}{\left|Q\right|}\sum_{i=1}^{\left|Q\right|}\frac{1}{rank_{i}},$$ (9)

where *Q* is the number of candidates around the target, and *rank*_*i*_ indicates the rank of the target in the output sample. In other words, the higher the rank, the higher the MRR value.

We have conducted the sensitivity analysis on the hyperparameters in the proposed method. Changing the parameters in the rules results in only a slight degradation in performance, whereas changing the number of layers and nodes in the deep learning model does not cause any difference in performance: top-1, 3, and 5 accuracies are 72.21%, 86.14%, and 88.88%. Table 3 shows the results on the sensitivity analysis, which confirms that the proposed method is not that sensitive to the change of the hyperparameters.

**Table 3:** Results on the sensitivity analysis.

	Baseline			Model w/ diff. hyperparameters		
	Top-1	Top-3	Top-5	Top-1	Top-3	Top-5
Average	73.76%	88.69%	91.54%	72.21%	86.14%	88.88%
Std. dev.	0.13	0.08	0.07	0.13	0.09	0.08
p-value	-	-	-	0.49	0.08	0.05

### 4.2 Results of mobility pattern analysis

**Fig 6:**
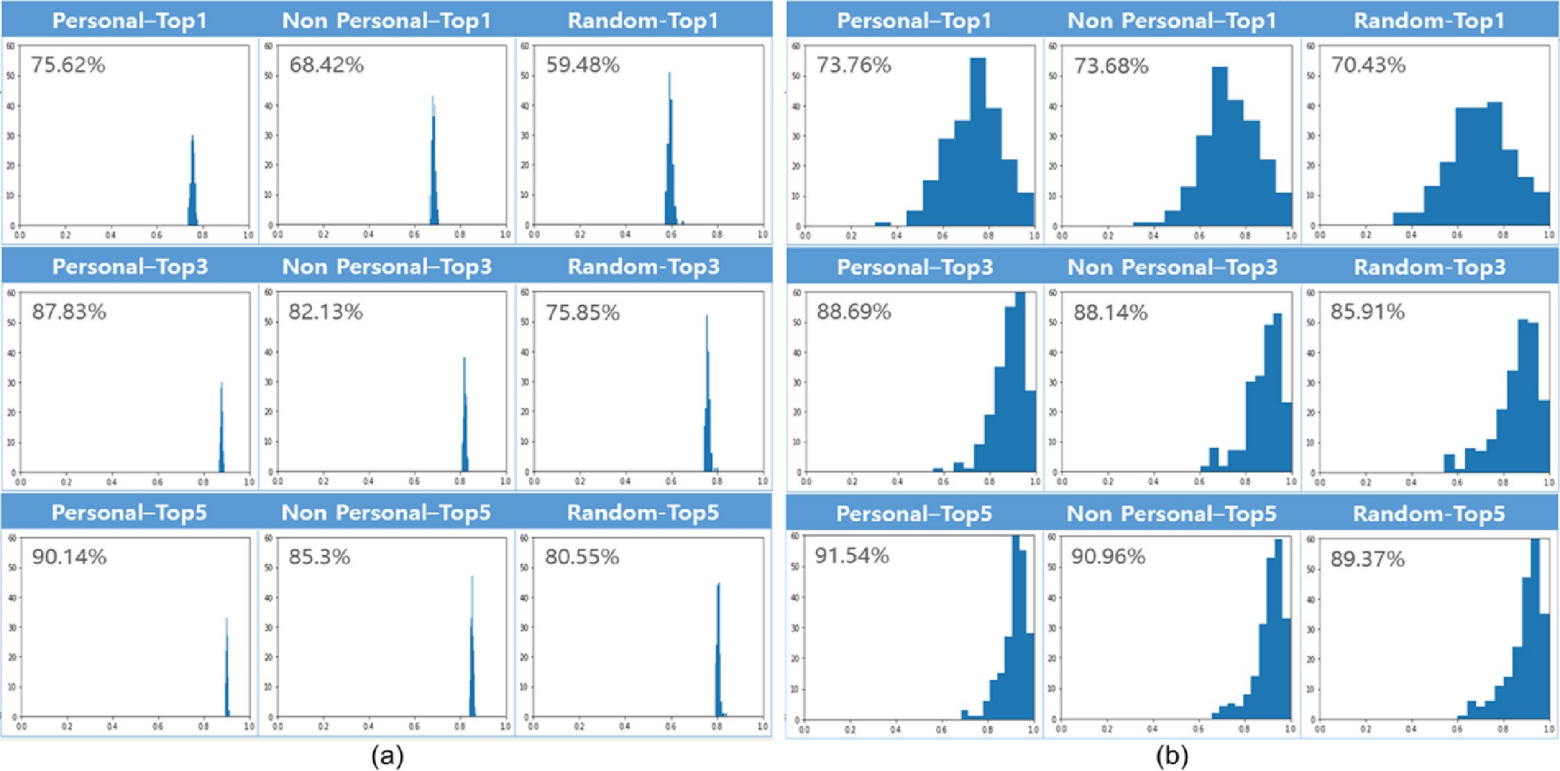
Prediction results using (a) random clustering and (b) mobility pattern clustering. We also present the prediction results for different embedding methods.

We conducted 10-fold cross-validation to evaluate the performance of the proposed model. We used basic features, personalized embedding, and resident vectors for successive POI prediction. Fig 6(A) presents the results of POI prediction using random clustering. The top-k accuracy of the personalized embedding method is higher than that of the other embedding methods by margins of 10% to 16%, which demonstrates that personalized embedding can provide more precise information for prediction models. Fig 6(B) presents the results of predicting the next POI using mobility pattern clustering. The results are similar to those in Fig 6(A), except for the top-one accuracy. However, the number of clusters with 70% accuracy is reduced and that of clusters with 80% accuracy is increased. Both sets of results demonstrate that our embedding and clustering method can improve the performance of predicting the next POI.

**Fig 7:**
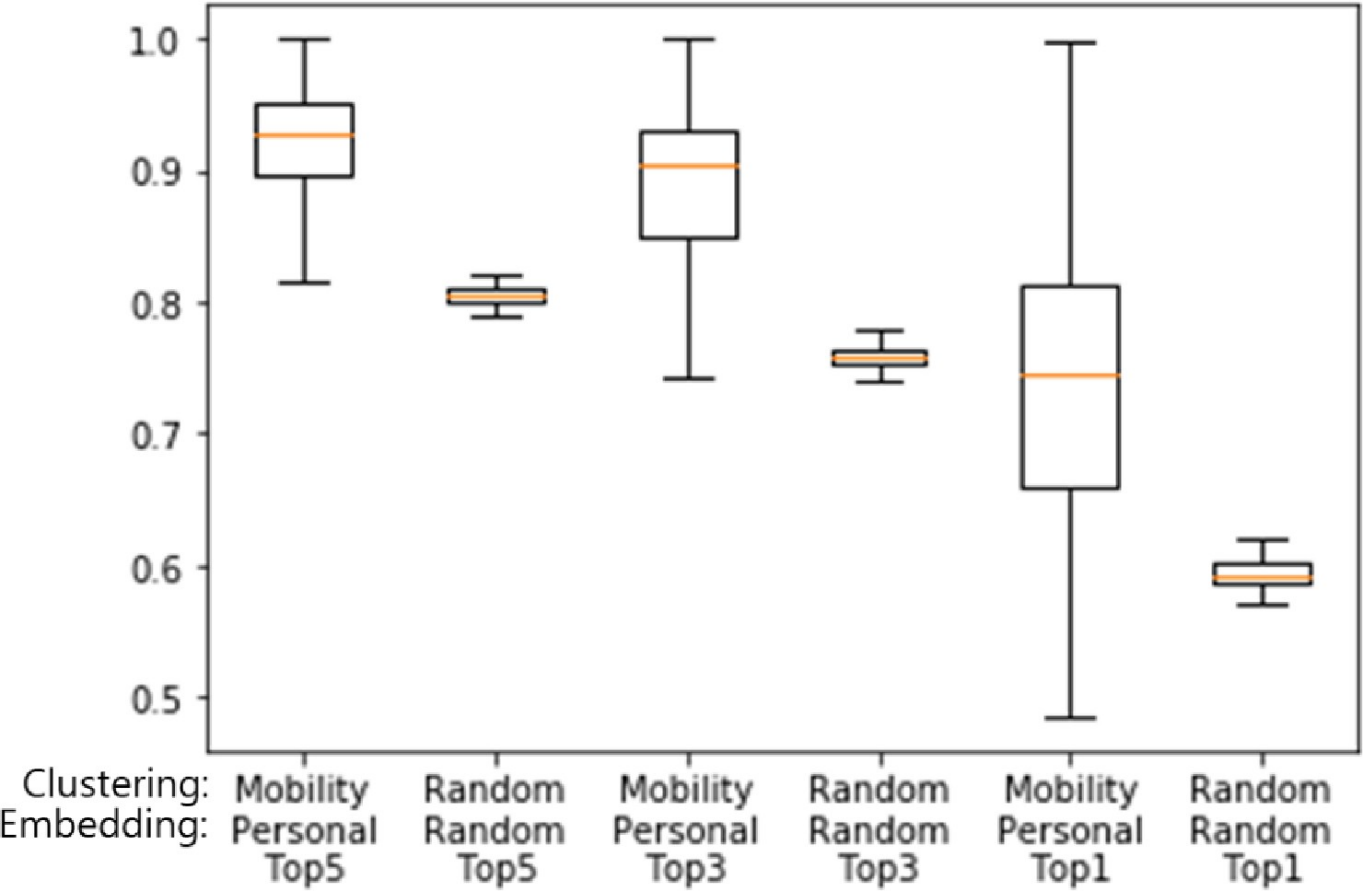
Results of 10-fold cross validation for verifying the performances of the proposed method and baseline methods. The y axis represents accuracy.

When random embedding is considered to determine if the proposed clustering method works well with any embedding model, the resulting top-1 accuracy is 59.48% for random clustering and 70.43% for mobility-based clustering. For the top-3 and top-5 accuracies, similar results can be observed, which confirms that our clustering method is effective. Fig 7 presents the distribution of accuracies in the form of box plots based on ten repetitions of our experiments. For a given prediction model, performance is improved when the embedding vectors are learned by the proposed method. Table 4 compares the results of all pairs of clustering and embedding methods. These results indicate that our methods reflect personal characteristics precisely, resulting in superior performance. One can see that there is an improvement in prediction performance when one of our methods is used. The proposed method always produces the better prediction performance with only one exception where the random clustering manages the diversity of the destination POI ID effectually. Even this case can be compensated by the proposed personalization method. To verify that there is a statistically significant difference, we present the results of a t-test between the baseline and proposed methods in Table 5. One can see that our results are statistically significant.

**Table 4:** Accuracy and standard deviation of each method.

Clustering method	Embedding method	Top-1	Top-3	Top-5
Mobility pattern clustering	Our personalization method	73.76±0.131	88.69±0.079	91.54±0.066
	Non-personal [21] + our clustering method	73.68±0.13	88.14±0.087	90.96±0.074
	Random	70.43±0.146	85.91±0.103	89.37±0.087
Random clustering	Our personalization method	75.62±0.01	87.83±0.005	90.14±0.005
	Non-personal [21]	68.42±0.009	82.13±0.007	85.3±0.006
	Random	59.48±0.013	74.85±0.01	80.55±0.008

**Table 5:** Test results for the proposed method and baseline method.

Type	Metric	Proposed method	Baseline
Top-1	Mean	0.7376	0.5948
	Standard deviation	0.0171	0.0001
	p-value	<0.05	
Top-3	Mean	0.8869	0.7585
	Standard deviation	0.0062	0.0001
	p-value	<0.05	
Top-5	Mean	0.9154	0.8055
	Standard deviation	0.0044	0.00007
	p-value	<0.05	

**Fig 8:**
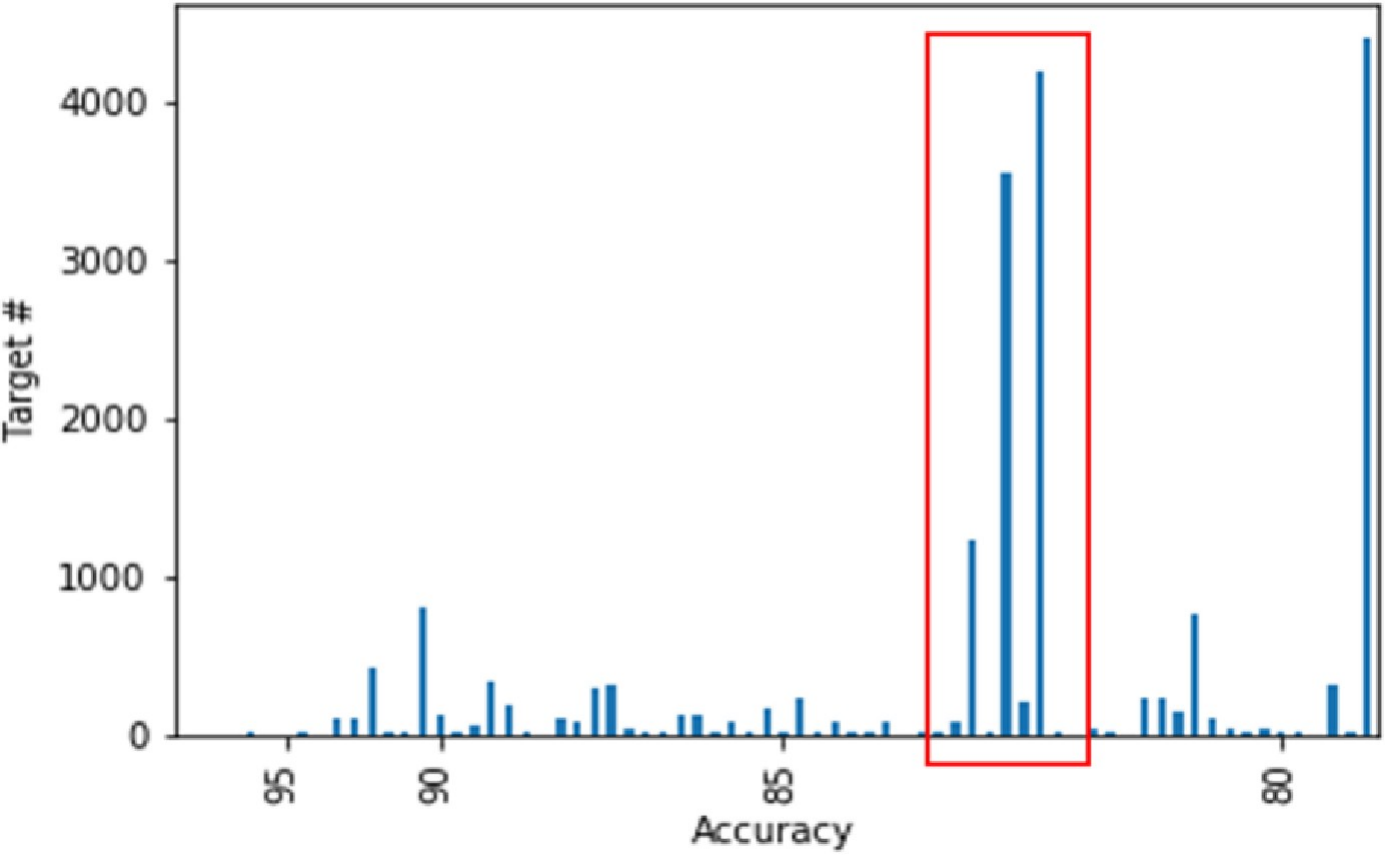
Number of targets in the model versus top-one accuracy.

Fig 8 plots the number of targets versus accuracy. Although there are clusters containing more than 4,000 targets and hundreds of residents, our method can predict successive POIs with an accuracy of 82%. This result indicates that small amounts of similar data are not ignored during clustering and that the characteristics of the individuals are reflected accurately in the embedding vectors and mobility patterns. This indicates that the proposed method models urban mobility accurately in complex environments based on the fact that mobility can be accurately predicted with more than 4,000 candidate destinations.

We further validate the proposed method by comparing with various machine learning algorithms such as decision tree (DT), random forest (RF), and naïve Bayes (NB) classifier. The hyperparameters of each model are set to default values in the scikit-learn library. Table 6 shows the results that the performance of the proposed method is much higher than that of random embedding. The number of next POI candidates to be predicted for each cluster is 249 on average, ranging from a minimum of 5 to a maximum of 3995. It turns out that the clustered embedding with MLP performs significantly better than other models.

**Table 6:** The results of random and clustered embedding methods with various predictors.

		DT	RF	NB	MLP
Random embedding	Top1	*6.65±3.39*	*7.47±3.92*	*2.82±1.70*	*75.62±0.01*
	Top3	*6.58±3.38*	*8.13±4.10*	*4.93±2.65*	*87.83±0.01*
	Top5	*6.50±3.36*	*8.49±4.23*	*6.25±2.95*	*90.14±0.01*
Clustered embedding	Top1	*20.33±0.26*	*21.19±0.27*	*14.32±0.22*	*73.76±0.13*
	Top3	*24.93±0.29*	*28.39±0.31*	*27.97±0.30*	*88.69±0.08*
	Top5	*29.46±0.31*	*32.71±0.32*	*34.01±0.34*	*91.54±0.07*

### 4.3 Validation of embedding vectors

We validated the information in the trained vectors in addition to prediction accuracy. Eqs (10) and (11) are used to determine whether the personalized mobility features are accurately reflected in the embedding vectors. The subtraction operation eliminates some information from a vector and the add operation attaches some information to a vector. In Eq (10), the first operation subtracts the “home” feature in the first operand. The second operation adds the “work” feature to the result of the first operation and the resident B information is offset, resulting in a vector containing resident A and “work” information. We can verify the POI class features using Eq (10). Eq (11) is used to verify resident information. As discussed by Mikolov et al. and Le [35, 36], we can compute the similarity between the properties of vectors using Eqs (10) and (11).

**Fig 9:**
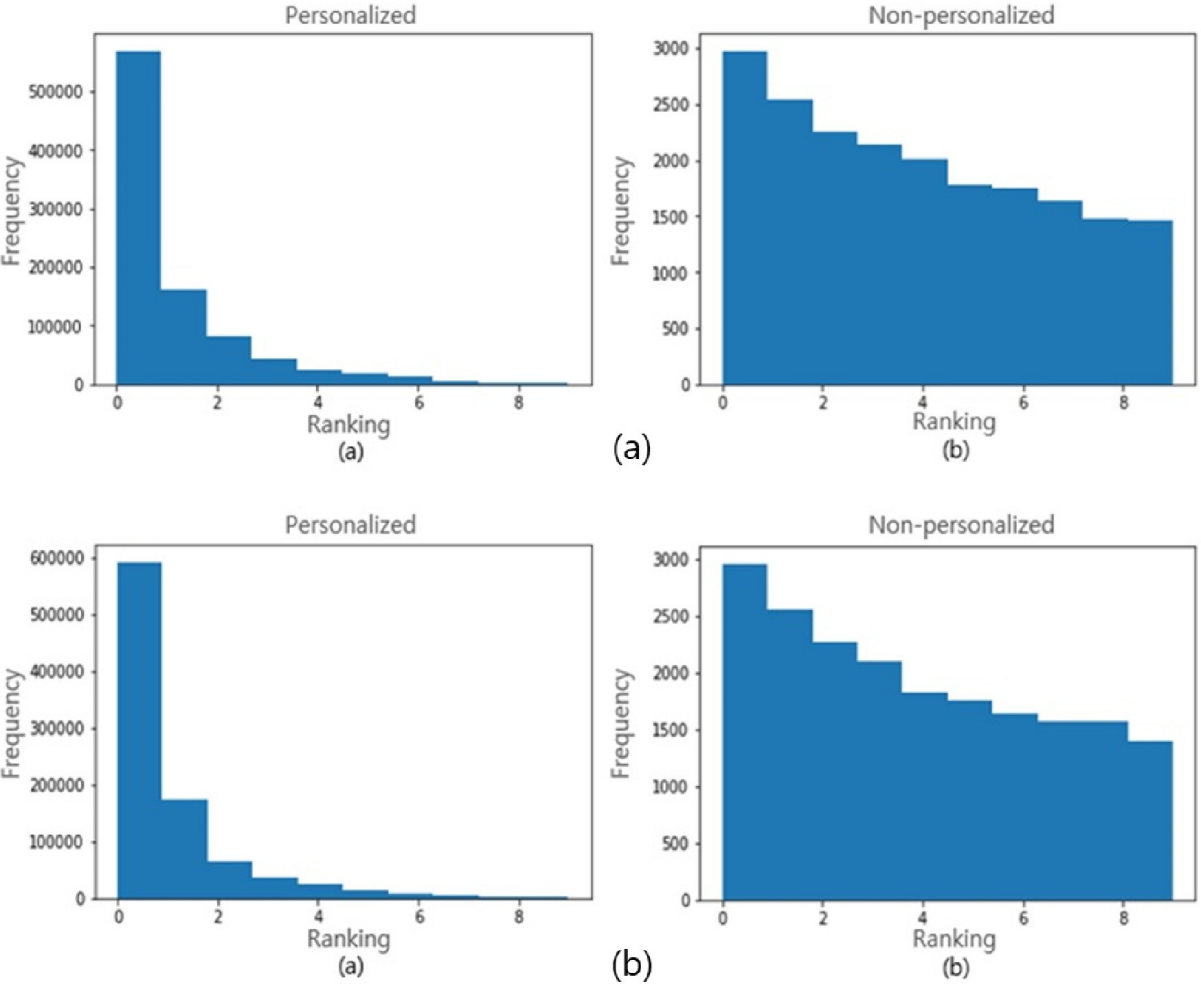
Ranking results for the target candidate for each embedding method with (a) Eq (10) and (b) Eq (11).

We randomly sampled 1,000 people from the 74,241 people in the dataset and then tested 990,000 cases of user combinations. We set the value of *Q* in Eq (9) to ten. Fig 9(A) presents the results of similarity testing based on Eq (10). For more than 80% of the samples, the similarity to the desired target falls in the first or second bin. However, when we use non-personalized embedding vectors, only a few thousand outputs fall in the first or second bins. This result demonstrates that our embedding vectors are valuable and reflect personal features accurately. Similar results can be observed in Fig 9(B) based on Eq (11). To evaluate these results quantitatively, we computed the MRR value, as shown in Table 7. The MRR for personalized embedding is 0.759 and that for non-personalized embedding is 0.349 according to Eq (10). These results represent an improvement of over 100%. Similarly, the results of Eq (11) reveal a significant improvement from 0.352 to 0.776. This indicates that the complexity highlighted in Fig 2 is embedded efficiently enough to capture the relevant relationships, even with vector arithmetic.

$${Resident}_{A,home}-{Resident}_{B,home}+{Resident}_{B,work}={Resident}_{A,work}$$ (10)

$${Resident}_{A,home}-{Resident}_{A,work}+{Resident}_{B,work}={Resident}_{B,home}$$ (11)

**Table 7:** MRR values for verifying the information in the mobility and resident embedding vectors.

	Proposed method	Baseline
Mobility feature	0.759	0.349
Resident feature	0.776	0.352

As a result, the vectors learned by the proposed model can provide significant information to the prediction model, which was already confirmed in the experiments discussed earlier.

## 5. Related works

Various methods for mobility pattern embedding and successive POI prediction have been presented. Most studies have attempted to extract the temporal and geographical influences of user movement sequences by using RNNs. An RNN can model serial data and POI prediction must capture some information in a serial movement sequence [37]. Zhao et al. modeled this information using three pairs of concepts (user-POI, POI-time, and POI-POI) and used it to identify interaction relationships using a pairwise tensor factorizing framework [18]. Cheng et al. proposed a factorization method personalized by a Markov chain (FPMC) [17]. They used a personalized Markov chain in their model, but they only used the relationships with previous POIs and made strong assumptions regarding various factors. Zhao et al. and Cheng et al. identified the relationships between users and POIs, but did not consider that a small amount of data might be ignored when training a model, meaning that they did not fully consider the individual mobility characteristics of users [17, 18]. Liu et al. pointed out the limitations of the strong assumptions of Markov chains (independent) and addressed the cold start problem in [17] and [18], respectively. These methods are ineffective at modeling continuous time and geographic impacts [17, 18]. To overcome this issue, Liu et al. proposed models based on spatiotemporal RNNs. Their model can reflect continuous temporal and geographical sequences [21]. Wang et al. attempted to learn embedding vectors for mobility based on similarity. The resulting sequence was used for training an RNN [38]. This method can represent a complete data sequence, but it requires additional temporal and geographical information and cannot reflect individual characteristics.

Some researchers have used methods to express the characteristics of movement sequences in a latent space. The Word2Vec method is widely used in many embedding models [16, 39]. The performance of the word embedding method has been verified in the field of natural language processing. Liu et al. considered movement sequences as sentences and embedded each mobility data sample as a word. They trained embedding vectors using a skip gram [21]. Liu et al. also used information regarding user preferences [21]. However, their method only reflects a portion of the user information because it only considers the top-n preferred POI data for capturing personal information. Feng et al. noted that previous studies failed to incorporate geographic influences [23]. They proposed a novel embedding method that considers geographical influences. Although they successfully incorporated geographical influences, they only considered preferences for reflecting personal characteristics [23]. Kang et al. expressed the characteristics of POIs in a latent space, similar to the method in [39]. However, Kang’s method embeds text information gathered from simple notification services [39]. This text information is used for evaluating mobility characteristics, but it also contains additional information, resulting in unnecessary overhead for data collection. Wang et al. used a knowledge graph for encoding semantic information, but this method depends heavily on how one constructs the knowledge graph. Although such a graph can accurately capture personal relationship information, the corresponding embedding technology is difficult to implement [40]. Zang et al. focused on geographical information. They attempted to encode personal POI preferences according to distance, but their method can only model personal geographical information, not temporal information.

Other researchers have attempted to obtain additional information regarding user mobility to improve performance [19, 41]. Yao et al. considered the temporal popularity of POIs and human behavior patterns over time [19]. They focused on the fact that there is a difference between behaviors on weekdays and weekends, which can reflect user mobility patterns, but not personal information. Unlike previous methods, our method can reflect personal information by using personal POI classes for clustering. Zhao et al. attempted to identify the characteristics of mobility by increasing the time resolution of movement sequences [41]. They analyzed the patterns on each day of the week and considered preferred ranking information. However, the target of their pattern analysis was an entire user dataset, meaning they lost the specific characteristics of individuals. Hossein et al. considered the importance of POIs in terms of obtaining POI characteristics, but they used general POI distinctions, meaning s that they did not consider the personal significance of POIs [42]. In contrast, our method retains all information regarding personal mobility while maintaining general features.

Table 8 summarizes the previous works discussed above. Some focus on the temporal or geographical information contained in user mobility data. For more detailed features, some researchers have attempted to provide personalized recommendations based on user preferences or to include deeper or additive information. However, they did not consider that the meanings of POIs may differ for each user. One previous method analyzed mobility patterns for prediction, but did not reflect the personal characteristics of patterns. In this paper, we proposed a novel method consisting of two components for personalized POI embedding and mobility-based clustering. These components were verified by predicting future POIs. Personalized embedding is a vectorization method that reflects individual characteristics that can be used as information for prediction. Clustering based on user mobility patterns is used to generate models that can reflect individual characteristics.

**Table 8:** Summary of related works.

Category	Author	Description
Factional spatiotemporal modeling	Cheng [17]	Factorize personalized Markov chains
	Zhao [18]	Pairwise tensor factorizing framework
Continuous spatiotemporal modeling	Yao [19]	Use temporal popularity and spatial-temporal human mobility
	Zhao [20]	Hierarchical geographical matrix factorization model
	Liu [21]	Spatial-temporal RNN
	Wang [38]	Similarity tree for organizing POI’s with an RNN
Adding personalized information	Liu [16]	Use the skip gram method and user top-n preference information
	Feng [23]	Incorporate geographical influences Propose a new method: POI2vec
	Baral [24]	Contextual POI sequence modeling using RNNs
	Wang [40]	Semantic contents are encoded via knowledge graph embedding
	Zhang [43]	Personalized geographical influence modeling method
Using specific information	Li [27]	Long-short-term-memory-based encoder-decoder framework
	Kang [39]	Embed POI sequences and text information
	Zhao [41]	Increase time resolution to capture more specific temporal characteristics from trip chains
	Hossein [42]	Incorporate sequential and categorical information from POIs

## 6. Conclusion

We proposed a novel method consisting of two main components (personalized mobility embedding and clustering based on mobility patterns) and verified these components by predicting successive POIs. Our method was verified using massive real-word data. Our dataset consists of 118 million movement sequences from 1.5 million users. It contains more than 15,000 target stations. In this data, we found that there is an imbalanced distribution with respect to target places and noted that this distribution is disadvantageous for users with a small number of data. To solve this problem, we proposed a novel personalized mobility embedding method that was verified through a similarity test. The results demonstrated that all data contain useful meta-information for predicting successive POIs. The prediction result demonstrated that our method improves performance and reflects mobility features accurately.

Because we cannot generate a model for every user for personalized recommendation, we proposed a clustering method based on mobility patterns and personalized embedding models. Our method can cluster similar users and represent individual characteristics. Experimental results confirmed that our clustering approach is useful for improving prediction performance. Our experiments revealed that it is effective to reflect individual information in mobility embedding vectors for predicting successive POIs. Even a simple prediction model yielded a high accuracy of 91.54% based on our embedding method. The results of a t-test demonstrated that our method yields statistically significant improvements, indicating that the complex patterns of urban mobility were effectively embedded, making it is easy to interpret the relationships between data. In the future, we will use more sophisticated models to exploit our embedding vectors and mobility patterns fully. We will also compare the proposed methods to other mobility embedding models. Furthermore, because our model’s accuracy is as high as 90%, we will construct a standalone system that can be applied in the real world.

## Supporting information

S1 Appendix Sharing the data. (DOCX)

## References

[1] Jiang R, Song X, Fan Z, Xia T, Chen Q, Miyazawa S, et al. DeepUrbanMomentum: An online deep-learning system for short-term urban mobility prediction. AAAI Conf. on Artificial Intelligence. 2018: 784–791.

[2] Liu Z, Li Z, Wu K, Li M. Urban traffic prediction from mobility data using deep learning. IEEE Network. 2018; 32(4): 40–46.

[3] Cascetta E, Pagliara F, Papola A. Governance of urban mobility: Complex systems and integrated policies. Advances in Complex Systems. 2007; 10: 339–354.

[4] Maggi E, Vallino E. Understanding urban mobility and the impact of public policies: The role of the agent-based models. Research in Transportation Economics, 2016; 55: 50–59.

[5] Bulkeley H, Betsill M.Rethinking sustainable cities: Multilevel governance and the urban politics of climate change. Environmental Politics. 2005; 14(1): 42–63.

[6] Kim JY, Lim KH, Cho SB. Personalized POI embedding for successive POI recommendation with large-scale smart card data. IEEE Int. Conf. on Big Data. 2019: 3583–3589.

[7] Adriansen HK, Nielsen TT. The geography of pastoral mobility: A spatio-temporal analysis of GPS data from Sahelian Senegal. GeoJornal. 2005; 64(11): 177–188.

[8] Liu L, Hou A, Biderman A, Ratti C, Chen J. Understanding individual and collective mobility patterns from smart card records: A case study in Shenzhen. IEEE Conf. on Intelligent Transportation Systems. 2009: 1–6.

[9] Ahas R, Aasa A, Silm S, Tiru M. Daily rhythms of suburban commuters’ movements in the Tallinn metropolitan area: Case study with mobile positioning data. Transportation Research Part C: Emerging Technologies. 2010; 18(1): 45–54.

[10] Gonzalez MC, Hidalgo CA, Barabasi AL. Understanding individual human mobility patterns. Nature. 2008; 453(7196): 779–782. 10.1038/nature06958 18528393

[11] Noulas A, Scellato S, Lambiotte R, Pontil M, Mascolo C. A tale of many cities: Universal patterns in human urban mobility. PLoS ONE. 2012; 7(5): e37027. 10.1371/journal.pone.0037027 22666339 PMC3362592

[12] Gallotti R, Bazzani A, Rambaldi S, Barthelemy M. A stochastic model of randomly accelerated walkers for human mobility. Nature Communications. 2016; 7(1): 1–7. 10.1038/ncomms12600 27573984 PMC5013551

[13] Liu Y, Wang F, Xiao Y, Gao S. Urban land uses and traffic ‘source-sing areas’: Evidence from GPS-enabled taxi data in Shanghai. Landscape Urban Plan. 2012; 106(1): 73–87.

[14] Zhong C, Arisona SM, Huang M, Batty M, Schmitt G. Detecting the dynamics of urban structure through spatial network analysis. Int. Journal of Geographical Information Science. 2014; 28(11): 2178–2199.

[15] Chen C, Chen J, Barry J. Diurnal pattern of transit ridership: A case study of the New York city subway system. Journal of Transport Geography. 2009; 17(3): 179–186.

[16] Liu X, Liu Y, Li X. Exploring the context of locations for personalized location recommendations. Int. Joint Conf. on Artificial Intelligence. 2016: 1188–1194.

[17] Cheng C, Yang H, Lyu MR, King I. Where you like to go next: Successive point-of-interest recommendation. Int. Joint Conf. on Artificial Intelligence. 2013: 2605–2611.

[18] Zhao S, Zhao T, Yang H, Lyu MR, King I. STELLAR: Spatial-temporal latent ranking for successive point-of-interest recommendation. AAAI Conf. on Artificial Intelligence. 2016: 315–321.

[19] Yao Z, Fu Y, Liu B, Xiong H, POI recommendation: A temporal matching between POI popularity and user regularity. IEEE Int. Conf. on Data Mining. 2016: 549–558.

[20] Zhao P, Xu X, Liu Y, Zhou Z, Zheng K, Sheng CS, et al. Exploiting hierarchical structures for POI recommendation. IEEE Int. Conf. on Data Mining. 2017: 655–664.

[21] Liu Q, Wu S, Wang L, Tan T. Predicting the next location: A recurrent model with spatial and temporal contexts. AAAI Conf. on Artificial Intelligence. 2016: 194–200.

[22] Wang MF, Lu YS, Huang JL. SPENT: A successive POI recommendation method using similarity-based POI embedding and recurrent neural network with temporal influence. IEEE Int. Conf. on Big Data and Smart Computing. 2019: 1–8.

[23] Feng S, Cong G, An B, Chee YM. Poi2vec: Geographical latent representation for predicting future visitors. AAAI Conf. on Artificial Intelligence. 2017: 102–108.

[24] Baral R, Li T, Zhu X. CAPS: Context aware personalized POI sequence recommender system. arXiv preprint arXiv: 1803.01245. 2018.

[25] Baral R, Li T. Maps: A multi aspect personalized POI recommender system. ACM Conf. on Recommender Systems. 2018: 281–284.

[26] Feng S, Li X, Zeng Y, Cong G, Chee YM, Yuan Q. Personalized ranking metric embedding for next new POI recommendation. Int. Joint Conf. on Artificial Intelligence. 2015: 2069–2075.

[27] Li R, Shen Y, Zhu Y. Next point-of-interest recommendation with temporal and multi-level context attention. IEEE Int. Conf. on Data Mining. 2018: 1110–1115.

[28] Elomre-Yalch R. A handbook: Using market segmentation to increase transit ridership. Transportation Research Board. 1988; 36.

[29] Ma X, Wu YJ, Wang Y, Chen F, Liu J. Mining smart card data for transit riders’ travel patterns. Transportation Research Part C: Emerging Technologies. 2013; 36: 1–12.

[30] Bhaskar A, Chung E. Passenger segmentation using smart card data. IEEE Trans. on Intelligent Transportation Systems. 2014; 16(3): 1537–1548.

[31] Lv Q, Qiao Y, Ansari N, Liu J, Yang J. Big data driven hidden Markov model based individual mobility prediction at points of interest. IEEE Trans. on Vehicular Technology. 2016; 66(6): 5204–5216.

[32] Mohamed K, Come E, Oukhellou L, Verleysen M. Clustering smart card data for urban mobility analysis. IEEE Trans. on Intelligent Transportation Systems. 2016; 18(3): 712–728.

[33] Ou Y, Cai C. Large-scale transit market segmentation with spatial-behavioral features. Transportation Research Parc C: Emerging Technologies. 2018; 90: 97–113.

[34] Craswell N. Mean reciprocal rank. Encyclopedia of Database. 2009: 1703–1703.

[35] Mikolov T, Chen K, Corrado G, Dean J. Efficient estimation of word representations in vector space. arXiv preprint arXiv: 1301.3781. 2013.

[36] Le Q, Mikolov T. Distributed representations of sentences and documents. Int. Conf. on Machine Learning. 2014: 1188–1196.

[37] Lipton ZC, Berkowitz J, Elkan C. A critical review of recurrent neural networks for sequence learning. arXiv preprint arXiv: 1506.00019. 2015.

[38] Wang MF, Lu YS, Huang JL. SPENT: A successive POI recommendation method using similarity-based POI embedding and recurrent neural network with temporal influence. IEEE Int. Conf. on Big Data and Smart Computing. 2019: 1–8.

[39] Chang B, Park Y, Park D, Kim S, Kang J. Content-aware hierarchical point-of-interest embedding model for successive POI recommendation. Int. joint Conf. on Artificial Intelligence. 2018: 3301–3307.

[40] Wang X, Salim FD, Ren Y, Koniusz P. Relation embedding for personalized translation-based POI recommendation. Pacific-Asia Conf. on Knowledge Discovery and Data Mining. 2020: 53–64.

[41] Zhao S, Zhao T, King I, Lyu MR, Geo-teaser: Geotemporal sequential embedding rank for point-of-interst recommendation. Int. Conf. on World Wide Web Companion Int. World Wide Web Conf. Steering Committee. 2017: 153–162.

[42] Rahmani HA, Aliannejadi M, Mirzaei Zadeh R, Baratchi M, Afsharchi M, Crestani F. Category-aware location embedding for point-of-interest recommendation. ACM SIGIR Int. Conf. on Theory of Information Retrieval. 2019: 173–176.

[43] Zhang Y, Liu G, Liu A, Zhang Y, Li Z, Zhang X, et al. Personalized geographical influence modeling for POI recommendations. IEEE Intelligent Systems. 2020.

