# Supplementary material for: Clustered embedding using deep learning to analyze urban mobility based on complex transportation data
Source: PLoS One. 2021 Apr 20;16(4):e0249318. doi: 10.1371/journal.pone.0249318 (PMC8057591; doi:10.1371/journal.pone.0249318)
Supplement: S1 Appendix — (DOCX) [file pone.0249318.s001.docx]

S1 Appendix. Sharing the Data

The mobility data used in this paper contain demographic information such as the user ID, date of birth, gender and GPS sequences. Because of the privacy issue, we cannot share the original data. Instead, and instead we share a subset of the anonymized data to replicate our study’s findings. The subset of the data is sampled by 'train_test_split' function with 'random_state' parameter as 30 from scikit-learn library in python. With this dataset, the proposed method achieves 73.35%, 86.52%, 89.07% in top-1/-3/-5 accuracy, which is 0.4%~2%p lower than that with the original data (73.76%, 88.69%, 91.54%), but still higher than the conventional method (68.42%, 82.13%, 85.30%). You can download the data in the form of comma-separated values (csv) at the *zonodo*:

http://doi.org/10.5281/zenodo.4309765
